# Supplementary material for: Experimental Evaluation of a Direct Fitness Effect of the De Novo Evolved Mouse Gene Pldi
Source: Genome Biol Evol. 2024 May 14;16(5):evae084. doi: 10.1093/gbe/evae084 (PMC11091481; doi:10.1093/gbe/evae084)
Supplement: evae084_Supplementary_Data [file evae084_supplementary_data.zip › SupplementaryFigures.pdf]

Experimental evaluation of a direct fitness effect of the *de novo* evolved mouse gene *Pldi*

Miriam Linnenbrink, Gwenna Breton, Pallavi Misra, Christine Pfeifle, Julien Y. Dutheil, Diethard Tautz

Supplementary Figures

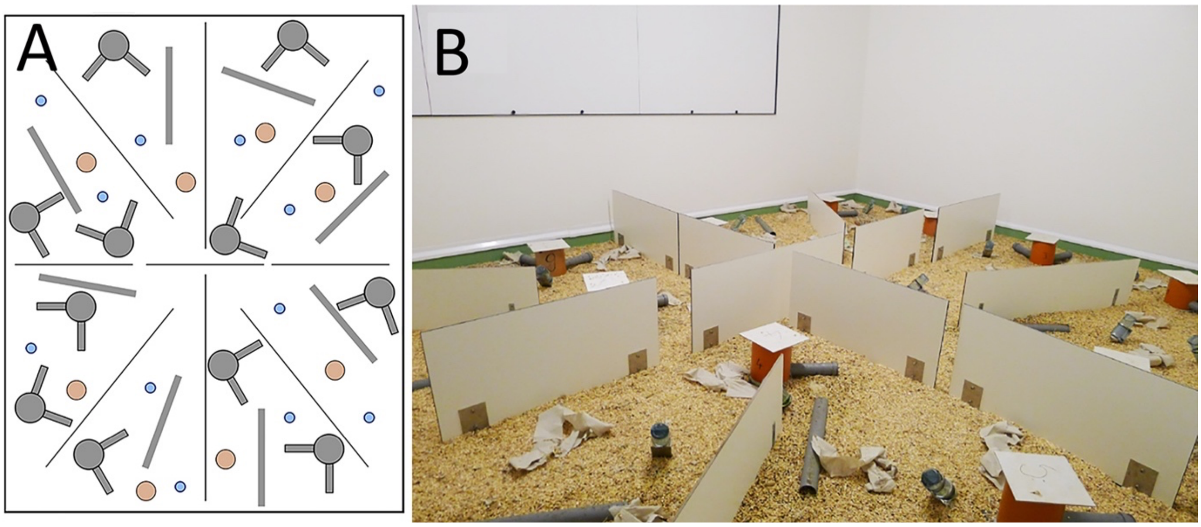

Supplementary Figure 1: Setup of the seminatural environment enclosures
A) Schematic representation of an enclosure. 4 quadrants were delimited with dividers. Each quadrant contained three houses with two tubes, three water bottles (blue circles), two feeding stations, and two additional tubes where mice could hide. B) View into an enclosure room.

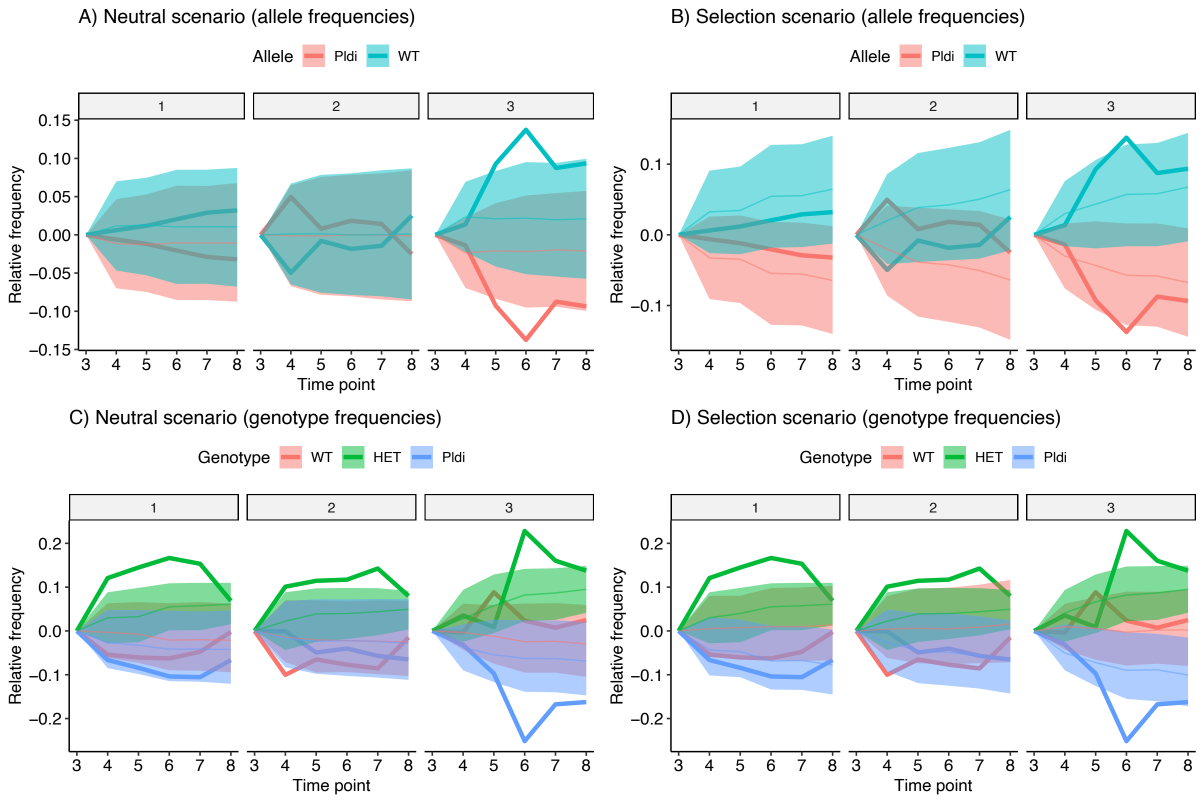

Supplementary Figure 2: Visualization of simulations overlapped with the actualy trajectories in the three rooms.

Top row (A and B): allelic frequencies. Bottom row (C and D): genotype frequencies. First column (A and C): neutral model. Second column (B and D): model with fitness effect in males. The shaded areas represent the trajectories of 100,000 replicates. The thick lines depict the trajectories observed in the experiment.

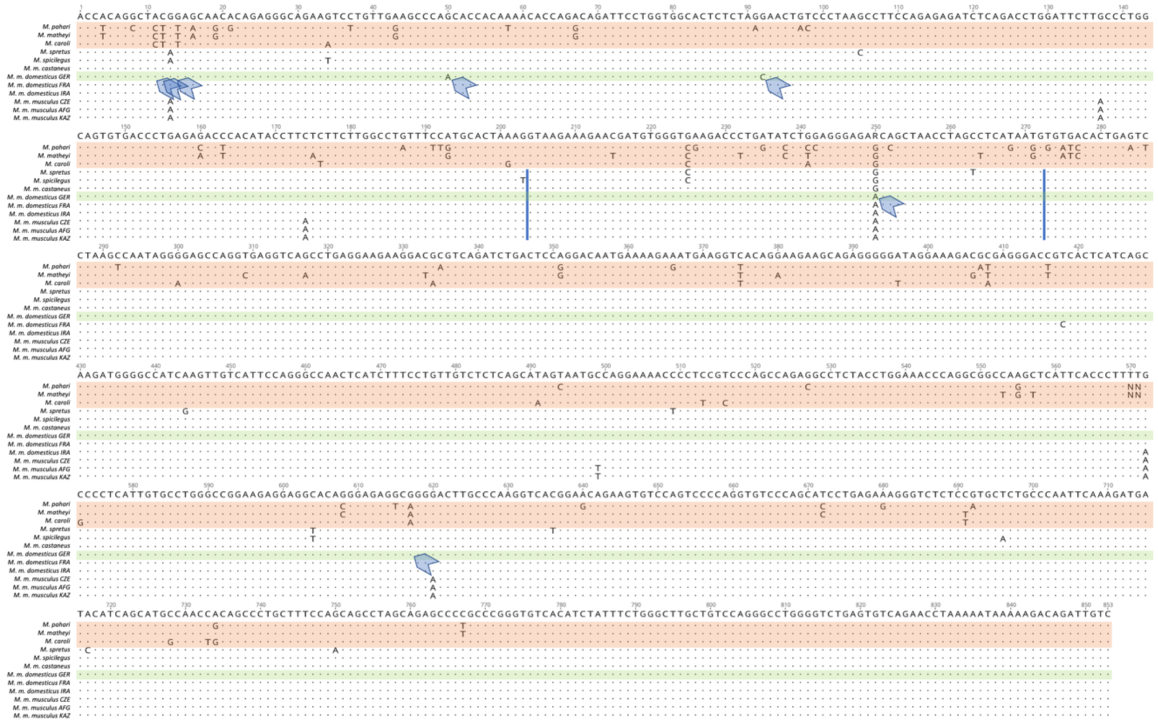

Supplementary Figure 3: Sequence alignments of the genomic regions coding for the *Pldi* RNA exons in *Mus* species and subspecies.

Outgroup species that do not express the *Pldi* RNA include *M. pahari*, *M. matheyi*, and *M. caroli* (shaded in red). The ingroup species are represented by population consensus sequences (obtained from the data described in (Harr et al., 2016) of *M. spretus*, *M. spicilegus*, as well as the subspecies *M. m. castaneus*, *M. m. domesticus*, and *M. m. musculus*. The latter two are represented by three populations each, labeled with 3-letter codes. The *M. m. domesticus* GER (shaded in green) corresponds to the *Mus musculus* reference sequence mm10, which is also the C57Bl6/J sequence that served here as the WT strain. Blue vertical lines indicate the positions of the introns. Blue arrows indicate substitutions in the *M. m. domesticus* GER sequence compared to the outgroups.
